# CT features of calcified micro-gastric gastrointestinal stromal tumors: a case series

**DOI:** 10.1186/s12880-023-01146-8

**Published:** 2023-11-20

**Authors:** Li-Jun Chen, Yue-Dong Han, Ming Zhang

**Affiliations:** 1 The Department for Radiology, Gao Xin Hospital Xi’an, Xi’an, Shannxi 710075 China; 2 Department of Medical Imaging, The First Affiliated Hospital of Xi’an Jiao Tong University, No.277, West Yanta Road, Xi’an, Shaanxi 710061 P.R. China

**Keywords:** Gastric stromal tumor, Calcification, Tomography, X-ray computer

## Abstract

**Background:**

Due to the lack of corresponding clinical symptoms, small calcified gastric gastrointestinal stromal tumors (GISTs) are often overlooked in clinical practice. Therefore, there is an unmet need to define the imaging features of calcified micro-gastric GISTs to facilitate diagnosis. This study retrospectively analyzed the computed tomography (CT) features of pathologically confirmed calcified micro-gastric GISTs.

**Methods:**

The medical records (gastroscopy, pre-treatment gastric CT imaging [pre- and post-contrast scans], pathology) of patients with calcified gastric GISTs < 1 cm in diameter confirmed pathologically after endoscopic submucosal dissection, endoscopic submucosal excavation, or endoscopic full-thickness resection were retrospectively reviewed.

**Results:**

Seven patients had 8 calcified gastric GISTs < 1 cm in diameter. Six patients hadsingle lesions, and 1patients had multiple lesions. Six patients had lesions in the gastric fundus, 1 patient had a lesion in the body of the stomach. Lesions had a mean diameter of 5.2 mm (range, 1.3 mm ~ 7 mm). Unenhanced CT scans showed spots and high-density nodular calcifications in 3 submucosal lesions, 2 lesions in the muscularis propria, and 3 subserosal lesions that protruded outside the stomach. Among the 8 lesions, only two had solid soft tissue components surrounding the calcification, with one of these two showing post contrast enhancement of the solid soft tissue component.

**Conclusions:**

Novel CT features of gastric GISTs included: commonly found in the gastric antrum, small size (< 1 cm in diameter), calcification, few solid soft tissue components, and no abnormal enhancement in most cases.

## Background

Gastrointestinal stromal tumor (GIST) is the most common mesenchymal tumor of the gastrointestinal tract. GISTs originate from the interstitial cells of Cajal [1], and most arise in the stomach. An estimated 20–50% of GISTs are malignant and associated with a high risk of recurrence and metastasis. These patients have a poor prognosis [2], and treatment requires curative surgical resection [3]. GISTs are discovered incidentally in 20% of cases, but symptoms may include gastrointestinal bleeding. Endoscopy, and endosonography when possible, are the diagnostic procedures of choice for small GISTs, while computed tomography (CT) is used for larger tumors [4].

On CT, GISTs are characterized by irregular morphology, a relatively large volume, necrosis, cystic changes, peripheral lymph node metastasis, and heterogeneous enhancement [5]. The calcification rate in gastric GISTs is approximately 21.6% [6]. Solitary or punctate calcification occurs in larger tumors [7, 8], prominent calcification is uncommon [9], and complete calcification in gastric GIST, especially in lesions < 1 cm in diameter, is exceedingly rare. Due to their small size, gastric GISTs < 1 cm in diameter are often overlooked in clinical practice. The objective of this study was to retrospectively analyze the imaging features on CT of calcified gastric GISTs < 1 cm in diameter confirmed pathologically after endoscopic submucosal dissection (ESD), endoscopic submucosal excavation (ESE), or endoscopic full-thickness resection (EFTR). Findings should increase awareness of these tumors among clinicians and inform clinical decision-making.

## Methods

### Patients

This study was approved by the ethics committee of Gao Xin Hospital Xi’an. All procedures were performed in accordance with the ethical standards of the institutional and/or national research committee and with the 1964 Declaration of Helsinki and its later amendments or comparable ethical standards.

Patients presenting with gastric submucosal lesions on endoscopy at Gao Xin Hospital between February 2018 and October 2020 were eligible for this study. Inclusion criteria were (1) available pre-treatment gastric CT imaging (pre- and post-contrast scans); and (2) underwent ESD, ESE, or EFTR with definite histopathological findings confirming calcified gastric GIST. Exclusion criteria were: (1) taking medications before the CT scan; or (2) patients who could not comply with the need to fast and drink water before the CT scan.

Medical records of included patients (*n* = 7) were retrospectively reviewed. Findings on gastroscopy, pre-treatment gastric CT imaging (pre- and post-contrast scans), and pathology were recorded.

### CT imaging acquisition

All patients underwent a 128-slice CT scan (Philips, Netherlands). Patients were required to fast for 4 ~ 6 h and drink 800 ~ 1000ml of water 30 min prior to undergoing upper abdominal examination. Scanning parameters were: 3-mm section thickness, 23-cm field of view (FOV), 256–256 matrix, 120 kV, 150–300 mA, screw pitch 0.98. Contrast enhanced CT scans used the non-ionic contrast agent Onipex, which was injected (2 ml/kg; flow rate 3ml/s) into the median cubital vein with a high-pressure syringe. Hepatic arterial phase and portal venous phase images were acquired with delays of 25–35 s and 60–70 s, respectively, after initiation of IV injection of contrast material, with delays of 3–5 min after initiation of IV injection of contrast material. The density, shape, distribution, and size of lesions were evaluated on all images by two associate chief physicians until consensus on imaging features was reached.

## Results

### Clinical data

This study included 7 patients (3 males and 4 females) with 8 calcified gastric GISTs aged between 51 and 78 years (median age, 59 years). All patients underwent gastroscopy due to abdominal pain, distension, acid regurgitation and belching. After gastroscopy revealed gastric submucosal lesions, CT scan was performed to further clarify the nature of the lesions and the status of the perigastric lymph nodes. Course of disease ranged from 2 days to 10 years. According to Chinese expert consensus on endoscopic diagnosis and treatment of digestive tract submucosal tumors, 2018 [10], all patients were indicated for endoscopic resection. No recurrence was observed in the 7 patients during 6 months to 1 year of postoperative follow-up. The clinical and CT characteristics of the study population are summarized in Table 1.

**Table 1:** Clinical and imaging characteristics of patients with gastric GIST (*n* = 7)

Baseline parameter	Variable
Age, years (range)	59 (51 ~ 78)
***Gender, n (%)***	
Male	3(42.8)
Female	4 (57.4)
***Location, n (%)***	
Fundus	6(85.7)
Body	1(14.2)
***Origin***	
Submucosal	3(42.8)
Muscle layer	2(28.5)
Subserosal	3(42.8)
***Growth pattern, n (%)***	
Endophytic	3(42.8)
Exophytic	3(42.8)
Mixed	1(14.2)
Size, mm, mean (range)	1.3 ~ 7(5.2)
***Heterogeneous enhancement***	
Yes	1(14.2)
No	6(85.7)

### Pathology

Diagnoses of calcified gastric GISTs were confirmed on pathology following ESD (3 patients), ESE (1 patient) and ESR (3 patients). Postoperatively, specimens were fixed with 4% neutral formaldehyde, embedded in paraffin, decalcified, sectioned, and stained with hematoxylin and eosin (HE). Gross observations revealed tumors were < 1 cm in diameter, gray-white, and hard. Tumors had a nodular shape with clear boundaries and no capsule. Microscopic observations revealed fusiform tumor cells arranged in bundles, little mitotic activity, and focal calcium deposits. Immunohistochemical findings are summarized in Table 2.

**Table 2:** Immunohistochemistry

CD117	7 (+)
CD34	7 (+)
DOG-1	5 (+)
H-Caldesmon	5(+)
SMA	7(-)
Des	7(-)
S-100	7(-)
Ki-67	≤2%
NIH grade	very low risk

NIH National Institutes of Health

Kits and antibodies were purchased from Maixin

### CT findings analysis

**Fig. 1:**
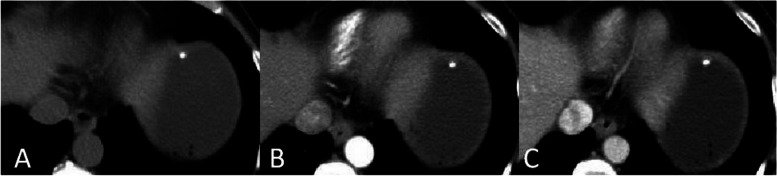
With a single submucosal stromal tumor. **A** Unenhanced CT scan showed nodular calcifications under the mucosa of the gastric fundus. CT scan in the arterial phase (**B**) and portal vein phase (**C**) showed the calcifications partially protruded into the gastric cavity, calcifications were surrounded by a few solid components, and no heterogeneous enhancement around the calcification foci

**Fig. 2:**
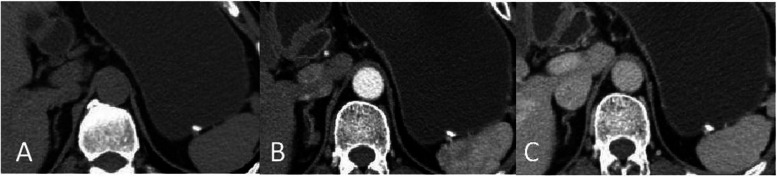
With a single submucosal stromal tumor. **A** Unenhanced CT scan showed that nodular calcifications in the submucosa of the gastric fundus partially protruded into the gastric cavity and there was a small amount of soft tissue, some of which protruded outside the stomach. CT scan in the arterial phase (**B**) and portal vein phase (**C**) showed heterogeneous enhancement of a small amount of soft tissue around the calcification and continuous enhancement of the portal vein phase

**Fig. 3:**
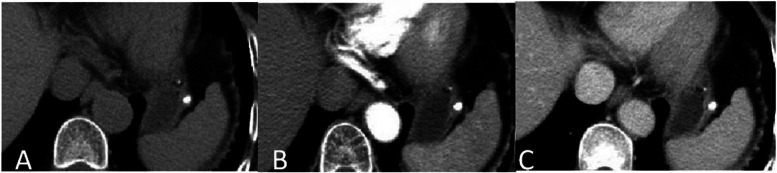
With multiple stromal tumors in the gastric fundus. **A **Unenhanced CT scan showed two nodular calcifications in the gastric fundus, with a smaller one located in the submucosal layer and a larger one located in the musculoserosal layer. CT scan in the arterial phase (**B**) and portal vein phase (**C**) showed no heterogeneous enhancement around the lesion

**Fig. 4:**
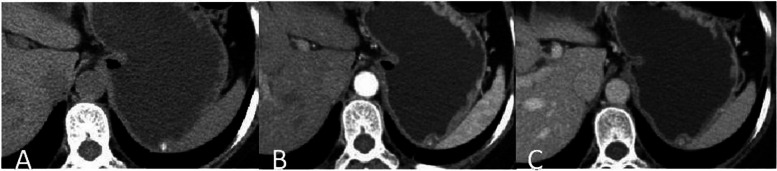
With a single stromal tumor in the gastric fundus. **A** Unenhanced CT scan showed nodular calcifications in the gastric fundus muscularis with no apparent soft tissue around them. CT scan in the arterial phase (**B**) and portal vein phase (**C**) showed no heterogeneous enhancement around the lesion, and a bulge in the adjacent mucosal layer

**Fig. 5:**
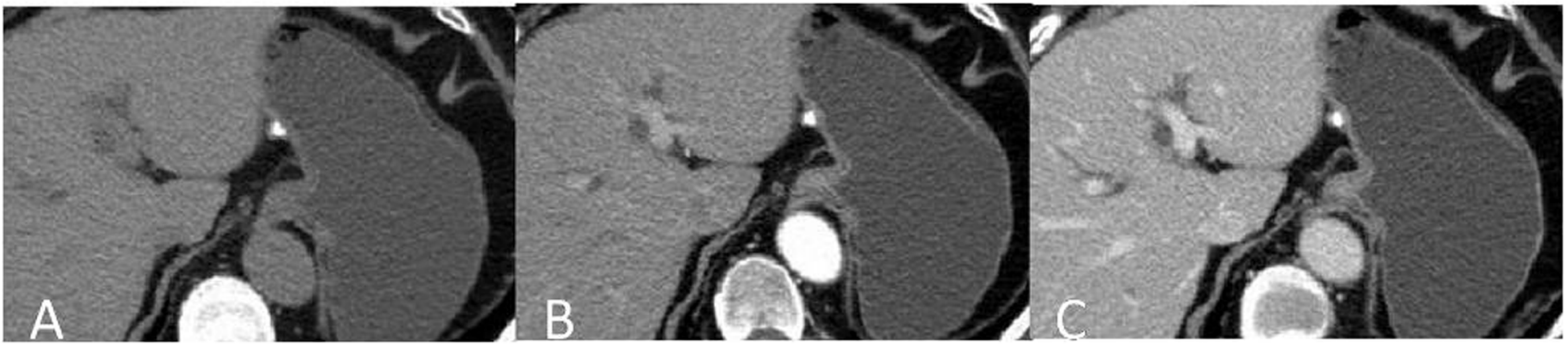
With a single stromal tumor under the serous membrane of the gastric fundus. **A** Unenhanced CT scan showed nodular calcifications in the gastric fundus subserosa protruding outside the stomach, with no apparent soft tissue around them. CT scan in the arterial phase (**B**) and portal vein phase (**C**) showed no heterogeneous enhancement around the lesion

CT scans from the 7 included patients demonstrated 8 calcified gastric GISTs. Six patients had a solitary lesion and 1 patient had multiple lesions. Six patients had lesions in the gastric fundus, and 1 patient had a lesion in the body of the stomach. Lesions had a mean diameter of 5.2 mm (range, 1.3 mm ~ 7 mm). Unenhanced CT scans of the 7 patients showed spots and high-density nodular shaped shadows, with CT values ranging from 230HU − 506HU. Out of 3 submucosal lesions, two had solid soft tissue components surrounding the calcification (Fig. 1A-C), with one of these two showing post contrast enhancement of the solid soft tissue component (Fig. 2A-C). Two lesions in the muscularis propria had corresponding thickening of the muscularis propria, bulged into the adjacent mucosal layer, and showed no enhancement (Figs. 3A-C and 4A-C). Three subserosal lesions protruded outside the stomach, did not appear to be surrounded by solid components, and showed no enhancement around the lesion (Fig. 5A-C). There were no metastatic lesions in the liver, peritoneum, or retroperitoneum on unenhanced or enhanced CT scans.

## Discussion

Under most circumstances, gastric GISTs are isolated tumors with an approximate diameter of 2 cm ~ 5 cm [11] and occasional amorphous calcifications [12]. Calcified GISTs usually occur in the stomach. On CT, these large tumors contain multiple large calcifications and have heterogeneous internal enhancement and solid components that may not be completely calcified [9, 13–15]. A literature search revealed no case reports describing an almost completed calcified gastric GIST with a diameter < 1 cm.

Gastric GISTs often exhibit an exophytic, intraluminal or mixed growth pattern. The origin of large gastric GISTs that have an extraluminal growth pattern may be difficult to identify [16]. In the present study, 3 submucosal lesions protruded into the gastric cavity, 2 lesions originating in the muscularis propria showed corresponding thickening of the muscularis and bulged into the adjacent mucosal layer, and 3 subserosal lesions protruded outside the stomach. The calcifications were surrounded by solid components in 2lesions. Seven patients underwent enhanced CT scans; of these, the solid components of 1 patient showed significant enhancement in the arterial phase, which persisted on delayed imaging. The other 6 patients showed no abnormalenhancement. Most patients had lesions in the gastric fundus. Six patients had single lesions, and 1 patient had multiple lesions, although previous reports of multiple calcified gastric GISTs are rare [17]. All lesions had an average diameter of 5.2 mm and were almost completely calcified. Calcification may occur in necrotic tissue [13], although the underlying mechanisms remain to be elucidated. In general, large rapidly growing lesions are more prone to necrosis. The association between necrosis, calcification and size of lesion requires further research.

Evidence suggests that CT features may be helpful for prognostic risk assessment in GISTs, and tumor size, morphology and growth pattern may be predictive of malignancy [18, 19]. According to the National Department of Health (NIH) risk classification system (2008) [20], the lesions in the present study were in the low-risk category as they had a diameter < 1 cm, and calcification cannot be used as a basis for risk classification [7, 21]. All GISTs expressed CD117 on immunohistochemistry, which may be present in both benign and malignant tumors [11].

Calcified gastric GISTs with a diameter < 1 cm should be differentiated from gastric leiomyoma, polyps, calcifying fibrous tumors and schwannoma. Gastric leiomyomas mainly occur in the gastric cardia, show an endoluminal growth pattern, and have less calcification than calcified gastric GISTs [22]. Gastric polyps usually occur in the lining of the gastric antrum, may be pedicled [23], and are larger and have less calcification than calcified gastric GISTs. Gastric calcifying fibrous tumors are small symptomatic tumors that occur in younger patients than those presenting with calcified gastric GISTs [24]. Pathological diagnosis is often required to distinguish between gastric calcifying fibrous tumors and calcified gastric GISTs. In schwannoma, calcification usually occurs in large regular tumors, with pronounced findings on enhanced CT [6].

This study was associated with several limitations. First, it was retrospective and included a small number of patients and lesions. Second, the lesions were small and contained few solid components that showed heterogeneous enhancement. Due to the small size of the lesions and the lack of definitive clinical features, most cases were discovered accidentally during gastroscopy and CT examinations that were performed due to upper abdominal discomfort. Lack of knowledge of these tumors meant calcifications were initially reported in only 3 of the 7 patients with lesions. In the remaining 4 patients, calcifications that protruded into the gastric cavity were mistaken for stomach contents. Due to the abnormal findings on conventional gastroscopy and CT, these patients did not undergo EUS; therefore, comparison with the results of EUS are lacking.

## Conclusions

In conclusion, this study identified novel CT features of gastric GISTs, including commonly found in the gastric antrum, small size (< 1 cm in diameter), calcification, fewsolid components, and noabnormal enhancement in most cases. These features may be particularly useful for identifying intracavital submucosal lesions. Although patients with micro or small GISTs rarely have symptoms and tumors seldom progress or metastasize, small calcified lesions of the stomach detected incidentally on CT should be evaluated with endoscopic ultrasound for further characterization and management/follow-up. Non-calcified micro-GISTs may go undetected on CT. Post-contrast enhancement is variable.

## Data Availability

The datasets generated and analyzed during the present study are available from the corresponding author on reasonable request.

## Abbreviations

CT: Computed tomography
GISTS: Gastric gastrointestinal stromal tumors
ESD: Endoscopic submucosal dissection
ESE: Endoscopic submucosal excavation
EFTR: Endoscopic full-thickness resection
